# Preparation, Characterization, and Bioactivities of Polysaccharide–Nano-Selenium and Selenized Polysaccharides from *Acanthopanax senticosus*

**DOI:** 10.3390/molecules29071418

**Published:** 2024-03-22

**Authors:** Xiaoli Li, Ying Li, Xueyan Wang, Rui Zhang, Jiaojiao Xue, Yi Ding, Xiuling Chu, Jianqing Su

**Affiliations:** College of Agronomy and Agricultural Engineering, Liaocheng University, Liaocheng 252000, China; lxl15006995983@163.com (X.L.); ly15963373832@163.com (Y.L.); wangxueyan202203@163.com (X.W.); m17863709708@163.com (R.Z.); x15065441211@163.com (J.X.); djy15373023047@163.com (Y.D.)

**Keywords:** *Acanthopanax senticosus*, selenium polysaccharide, polysaccharide–nano-selenium, antioxidant

## Abstract

*Acanthopanax senticosus* polysaccharide–nano-selenium (ASPS-SENPS) and *A. selenopanax* selenized polysaccharides (Se-ASPS) were synthesized, and their characterization and biological properties were compared. The acid extraction method was used to extract the polysaccharides of *A. selenopanax*, followed by decolorization using the hydrogen peroxide method and deproteinization based on the Sevage method, and the purification of *A. senticosus* polysaccharides (ASPS) was carried out using the cellulose DEAE-52 ion column layer analysis method. An *A. senticosus* polysaccharide–nano-selenium complex was synthesized by a chemical reduction method using ASPS as dispersants. The selenization of polysaccharides from *A. selenopanax* was carried out using the HNO_3_-Na_2_SeO_3_ method. The chemical compositions, scanning electron microscopy images, infrared spectra, and antioxidant properties of ASPS-SENPS and Se-ASPS were studied, and they were also subjected to thermogravimetric analysis. The results indicated that the optimal conditions for the synthesis of ASPS-SENPS include the following: when ASPS accounts for 10%, the ratio of ascorbic acid and sodium selenium should be 4:1, the response time should be 4 h, and the reaction temperature should be 50 °C. The most favorable conditions for the synthesis of Se-ASPS were as follows: m (Na_2_SeO_3_):m (ASPS) = 4:5, response temperature = 50 °C, and response time = 11.0 h. In the in vitro antioxidant assay, when the mass concentration of Se-ASPS and ASPS-SENPS was 5 mg/mL, the removal rates for DPPH free radicals were 88.44 ± 2.83% and 98.89 ± 3.57%, respectively, and the removal rates for ABTS free radicals were 90.11 ± 3.43% and 98.99 ± 1.73%, respectively, stronger than those for ASPS. The current study compares the physiological and bioactivity effects of ASPS-SENPS and Se-ASPS, providing a basis for future studies on polysaccharides.

## 1. Introduction

The medicinal components of *Acanthopanax senticosus* encompass the desiccated stems and rhizomes of *A. senticosus* plants, predominantly found in the forests and thickets of Northeast China’s Jilin province. The pharmaceutical composition of *A. senticosus* comprises polysaccharides, saponins, polyphenols, and various other compounds, which exhibit the therapeutic properties of replenishing qi to invigorate the spleen, nourishing the kidneys, and promoting mental tranquility.

Selenium is a necessary trace element for the body to be able to function. It is a very important element for animals and plants. It is widely involved in life activities in nature. Moderate selenium intake can provide increased antioxidant activity and lower the risk of cancer [1,2,3]. Nevertheless, the human body does not synthesize selenium, so humans have to rely on external sources to obtain it. Inorganic Se can be used within a narrow safe range in living organisms and can easily cause toxicity due to overdosing [4]. Among the organic selenides, selenium polysaccharides have low toxicity, are easily absorbed, and have significant biological activity.

Studies have shown that selenium polysaccharides show higher activity than polysaccharides. Luo et al. [5] prepared *Phellinus igniarius* selenium-enriched mycelia polysaccharides and investigated their antioxidant capacity and anti-inflammatory effects. The results showed that the *Phellinus igniarius* selenium-enriched mycelia polysaccharides reduced reactive oxygen species (ROS) levels, myeloperoxidase (MPO) activity, and malondialdehyde (MDA) content and increased the enzyme activities of glutathione peroxidase (GSH-Px) and catalase (CAT), showing a significant wound-healing effect in vivo, with no obvious cytotoxicity. Qiu et al. [6] successfully prepared *Lycium barbarum* selenium polysaccharides by using the nitric acid–sodium selenite method, studied their antioxidant capacity, and finally found that the antioxidant capacity of *Lycium barbarum* polysaccharides modified by selenium can be significantly higher; Gao et al. [7] successfully prepared alfalfa root selenated polysaccharides using nitric acid–sodium diselenite and compared their antioxidant capacity using ascorbic acid as a comparison. The experimental results indicated that the selenium-modified *alfalfa* root polysaccharides had more substantial antioxidant capacity. Zheng GAO et al. [8] successfully modified a *Vibrio radiculitis mycelium* polysaccharide by selenization using the HNO_3_-Na_2_SeO_3_ method and also studied its in vitro antioxidant effect and anti-inflammatory and lung protection properties in a pulmonary mice model. It was found that the antioxidative ability of the *mycelium* polysaccharide of Vibrio radiculoides was significantly improved after selenium modification.

Selenium nanoparticles (SeNPs) have received much attention due to their good bioavailability and very low virulence. However, the stability of SeNPs needs to improved. Polysaccharides, as a class of biomolecules, can play a good role in the construction of selenium nanoparticles due to their particular structure of suspension, emulsification, and stabilization [9], reducing the free energy on the surface of selenium nanoparticles, effectively preventing the aggregation of selenide nanoparticles and the reunion of nano-selenium particles and improving the biocompatibility of nano-selenium. This is the best feedstock for the preparation of selenium nanocomplexes. The methods used for the production of selenium nanoparticles mainly include physical, carrier template, and ultrasonic chemical methods [10]. Nano-selenium prepared by physical methods can control the particle size by adjusting the laser parameters, and is not easily contaminated by chemical reagents, but it is not widely used due to its low preparation efficiency and yield; the chemical method usually prepares nano-selenium by a redox reaction between sodium selenite and reducing agents, but the generated nano-selenium products are unstable and relatively low in biological activity. The carrier template method uses active groups to adsorb and wrap the selenium nanoparticles to prevent the aggregation and sinking of selenium nanoparticles, which is a commonly used preparation technique, and proteins [11], polymers [12], polysaccharides [13], etc., can be used as carrier templates to prepare selenium nanoparticles.

Combining selenium with polysaccharides to make organic selenium compounds, which maintains the basic configuration and biological activity of polysaccharides, improves bioavailability and reduces toxicity and adverse reactions. In this study, Se-ASPS and ASPS-SENPS were prepared from *A. senticosus* as raw materials, and their characteristics were compared to provide a theoretical basis for the further study of *A. senticosus* polysaccharides (ASPS) as medicine and performance foods.

## 2. Results and Discussion

### 2.1. Extraction and Purification of ASPS

**Figure 1:**
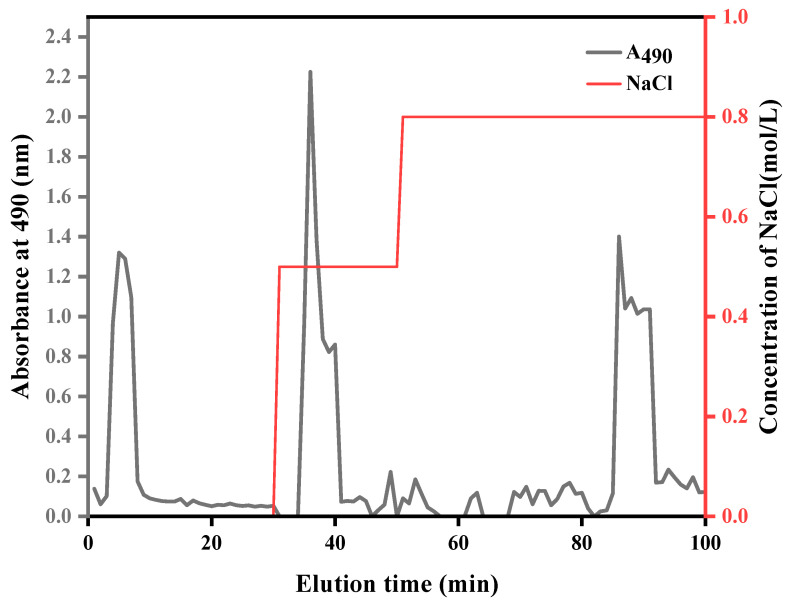
Polysaccharide elution curves.

The polysaccharides were extracted by acid extraction, precipitated by 75% ethanol, and oven-dried at 65 °C. A brownish powder was obtained with an extraction rate of 8.6%. The DEAE column method was used to purify the polysaccharides, and the elution curves are depicted in Figure 1: This method can separate two polysaccharides. The first peak eluted with double-distilled water (ddH_2_O) was a neutral polysaccharide; it was eluted with 0.5 mol·L^−1^ NaCl. A leading peak with a similar shape, good symmetry, and sharp shape appeared in the elution to 330–420 mL, which was the first acidic polysaccharide; gradient analysis was conducted by elution with 0.8 mol·L^−1^ NaCl solution. A polysaccharide absorption peak began to appear at 790 mL, which was the second acrid polysaccharide.

### 2.2. Preparation of A. senticosus Polysaccharide–Nano-Selenium

**Figure 2:**
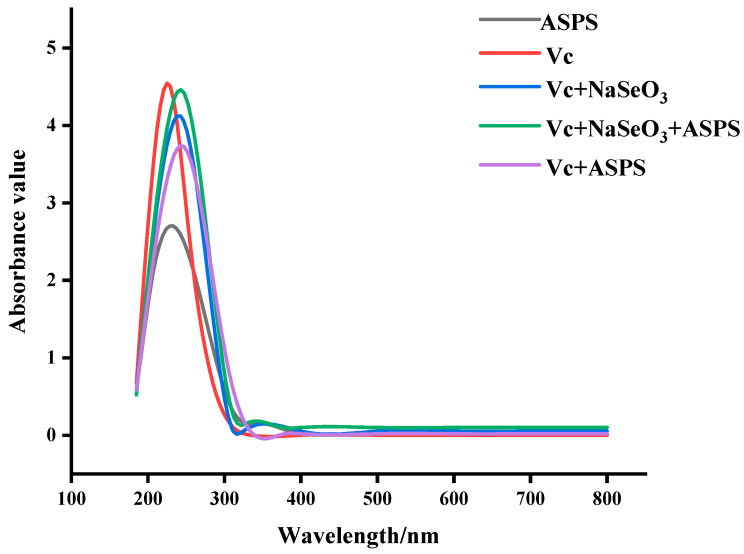
Ultraviolet absorption spectra of different solutions.

We pipetted 20 mL of 0.1 mol/L Vc solution in a conical bottle, added 10 mg/mL of ASPS solution, and then added 10 mL of 0.05 mol/L^−1^ of sodium selenite (Na_2_SeO_3_) solution. The conical bottle was then shaken well and placed in a constant-temperature water bath. The influence of process variables on the synthesis of ASPS-SENPS was investigated by varying the dosage of ASPS, the response time, the reaction temperature, and the proportion of ascorbic acid and sodium selenite (Figure 2). The A_410_/A_490_ values were also chosen to characterize the particle size of *A. senticosus* polysaccharide–selenium nanoparticles complexes based on the two-wavelength colorimetric method for colloidal solutions.

**Figure 3:**
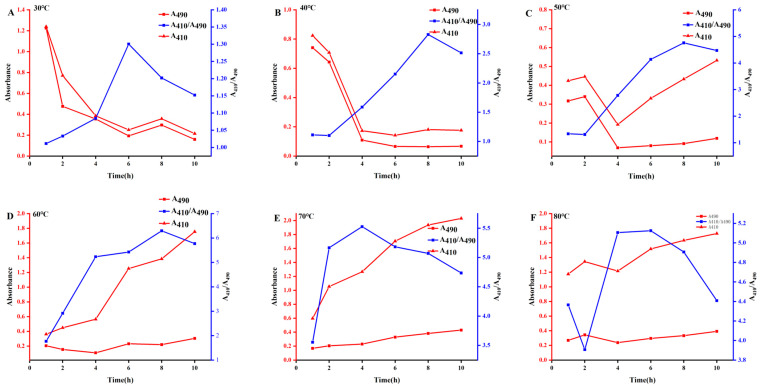
Effect of reaction time and reaction temperature on the preparation of complexes of polysaccharides and selenium nanoparticles. Note: (**A**–**F**) Effect of increasing the reaction temperature in the range of 30–80 °C and the reaction time from 1 h to 10 h on the synthesis of Acanthopanax polysaccharide-nanoselenium complexes.

As can be observed in Figure 3, the A_410_/A_490_ results of ASPS-SENPS all showed an overall increasing and then decreasing trend with time at different reaction temperatures, with the most significant ratio at 60 °C, eighth. It can be observed that the ASPS-SENPS prepared at this time have the smallest and most uniform and stable particle size [14].

**Figure 4:**
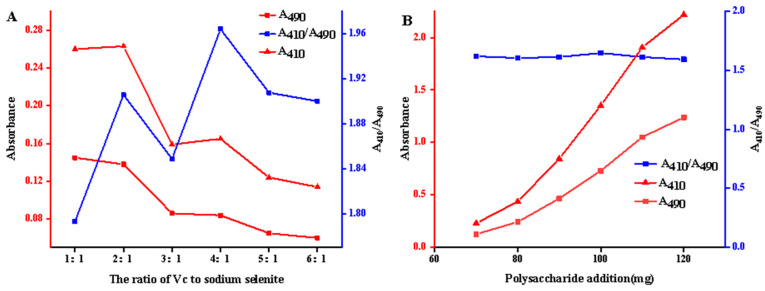
Effects of ascorbic acid and sodium selenite ratios and polysaccharide additions on polysaccharide-nanoselenium selenium complexes of *Acanthopanax senticosus*. Note: (**A**) shows the effect of the ratio of ascorbic acid and sodium selenite on the polysaccharide-nanoselenium complexes of *Acanthopanax senticosus*; (**B**) shows the effect of polysaccharide addition on the polysaccharide-nanoselenium complexes of *Acanthopanax senticosus*.

Figure 4A shows that when the ratio of asphatic acid to sodium selenite was 4:1, the A_410_/A_490_ value was the maximum. It has been shown that vitamin C can provide a reducing system in the system of preparing selenium nanoparticles, which makes the reaction proceed continuously in the direction of generating ASPS-SENPS and helps the generation of selenium nanoparticles, so 4:1 was chosen as the best reaction ratio.

From Figure 4B, it can be observed that the A_410_/A_490_ values of ASPS-SENPS showed a rising and then decreasing trend with the increase in polysaccharide addition. The A_410_/A_490_ values were the highest when the expansion of ASPS was 100 mg, indicating that the prepared ASPS-SENPS products were homogeneous and stable at this time.

### 2.3. Preparation of Selenium Polysaccharides of A. senticosus

**Figure 5:**
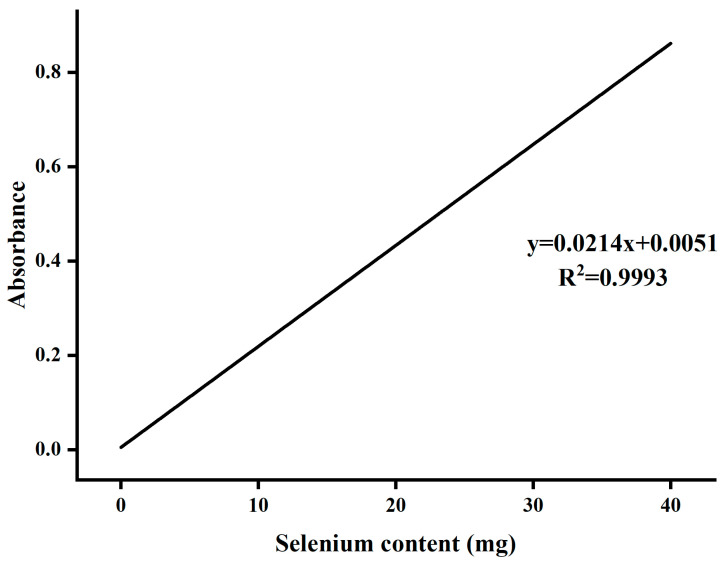
Analysis curve of selenium content.

The standard curve for determining selenium content is shown in Figure 5.

The modification conditions and selenium content of Se-ASPS are shown in Table 1. Among the nine kinds of sASPS, sASPS_1_, the first kind of *A. senticosus* selenium polysaccharide, had the highest selenium content, reaching 145.78 mg/g, followed by sASPS_2_ and sASPS_3_. The highest carbohydrate content belonged to sASPS_1_ (80.73%), followed by sASPS_5_ and sASPS_9_.

The chemical composition analysis of *A. senticosus* polysaccharides before and after selenization modification is shown in Table 2. The highest content of selenium was found in the Se-ASPS (145.78 mg/g), followed by ASPS-SENPS; the highest content of protein belonged to ASPS-SENPS, followed by ASPS (4.30%); ASPS-SENPS had the highest uronic acid content with 3.50%; the highest content of polysaccharide belonged to the Se-ASPS (17.12 mg/g); the Se-ASPS had the highest reducing sugar content, 49.36%.

### 2.4. UV Scan Results

**Figure 6:**
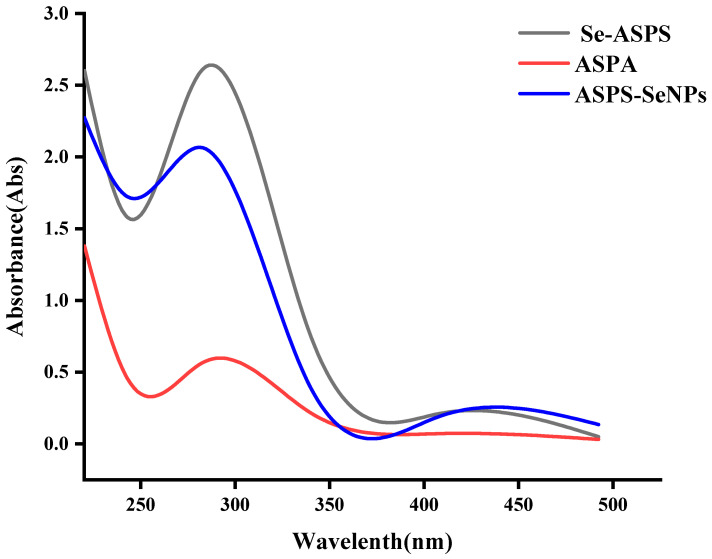
UV test.

More and more scholars are contributing to the development of selenopolysaccharides, which have broad prospects for application [15,16]. The method reported by Yunshan Zhang [17] was used to modify ASPS to obtain selected Se-ASPS. The determination of the purity of the Se-ASPS was carried out by UV-vis chromatography, and the results are displayed in Figure 6. The absorption peaks were found in aqueous solutions of ASPS and Se-ASPS at 260 nm and 280 nm, indicating that the ASPS and Se-ASPS contained nucleic acids and proteins. ASPS-SENPS were prepared by Jiao [9], and their purity was measured by UV-vis chromatography, and the results are presented in Figure 6, with absorption peaks at 260 nm and 280 nm, indicating that ASPS-SENPS, like ASPS and Se-ASPS, both contain nucleic acid and protein.

### 2.5. Electron Microscope Observation

**Figure 7:**
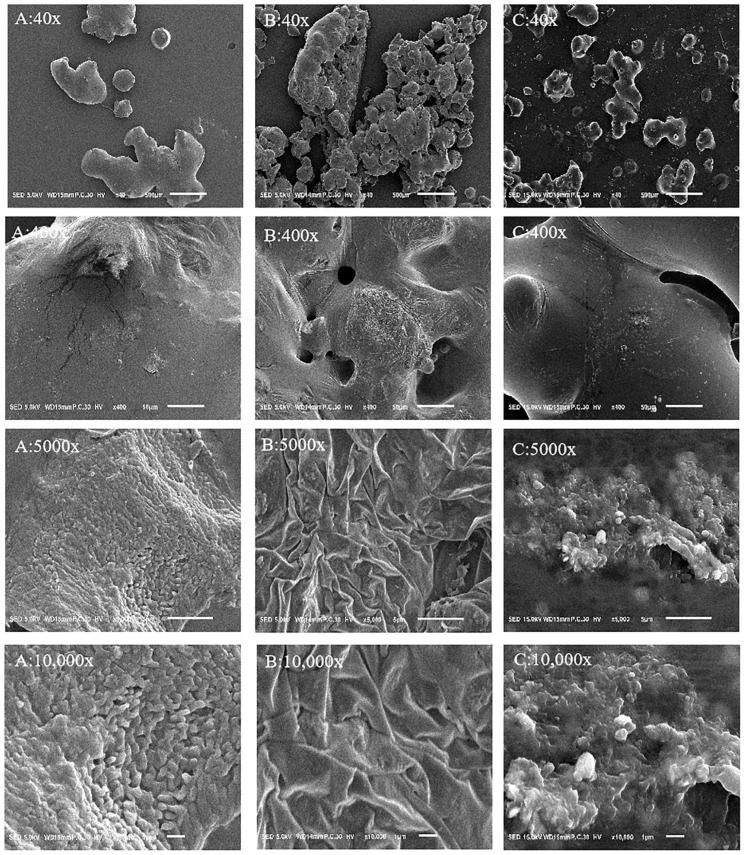
Electron microscope observations at different magnifications: (**A**) electron microscope observation of ASPS; (**B**) electron microscope observation of Se-ASPS; (**C**) electron microscope observation of ASPS-SENPS.

Figure 7 shows magnified SEM images of the three polysaccharide examples, from 40 to 10,000 times magnification. The SEM images of ASPS at 40, 400, 5000, and 10,000 magnification showed a smooth surface with a compact and irregular spherical structure, and the spherical structure increased with increasing magnification, with a non-uniform distribution of smooth spherical particles (as shown in Figure 7A). The Se-ASPS, on the other hand, increasingly showed how their structure is different from that of ASPS with increasing magnification, showing a dense stripy and flakey appearance, along with a broken and folded but relatively smooth surface (as shown in Figure 7B), suggesting that selenide modification has a remarkable effect on the surface topography of ASPS; compared with the electron microscopy images of ASPS, the structure of ASPS-SENPS is denser, and the shape is closer to a spherical shape, showing that the ASPS-SENPS showed more monodisperse and homogeneous particles (as shown in Figure 7C), indicating that the ASPS modified surface could effectively inhibit the mutual aggregation between nanoparticles and increase their stability.

### 2.6. Thermogravimetric Analysis

**Figure 8:**
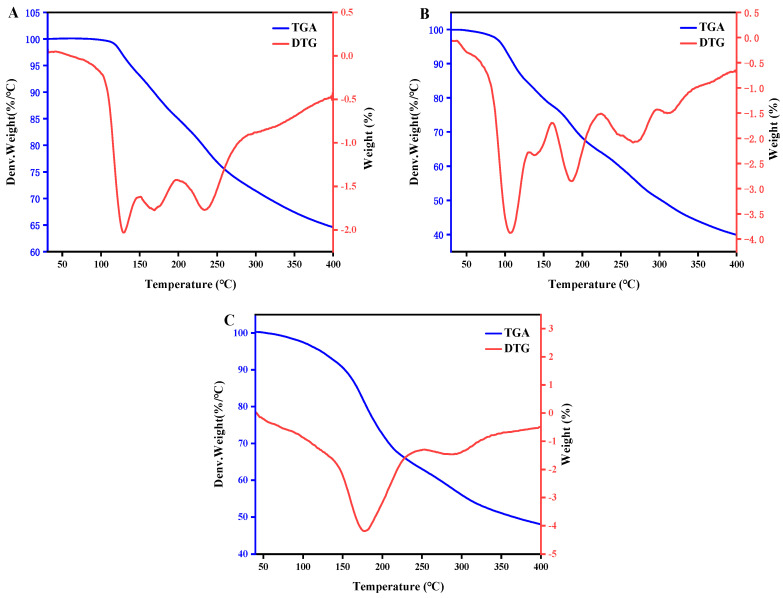
Thermogravimetric and differential thermal analyses for a heating speed of 10 °C/min. (**A**) TGA and DTG profiles of ASPS; (**B**) TGA and DTG profiles of Se-ASPS; (**C**) TGA and DTG profiles of ASPS-SENPS.

The thermal gravimetric curve of ASPS is shown in Figure 8. For the first two polysaccharides, the degradation process was categorized into four stages. Regarding ASPS, the first phase of weight loss was in the range of 10–150 °C, mainly attributed to the weight loss of free and bound water, and the mass loss rate was about 6.94%, with the maximum peak at 128.9 °C; the second phase of value loss appeared in the range of 150–205 °C, with the value of 8.51%, which may be associated with the thermal breakdown of polysaccharides; the third phase of value loss was in the range of 205–285 °C, with a gradual decrease in mass due to the thermal decomposition of carbon, meaning that its mass gradually decreased; the fourth weight loss range occurred above 285 °C, and the weight loss was 8.31%.

For Se-ASPS, the first stage weight loss range is 10–160 °C, mainly due to the loss of both free and confined water, with a mass loss rate of about 21.99% and a peak of 107.7 °C at a rate of 3.88%/min. The second weightlessness phase occurs at 160–225 °C, with a weightlessness value of 14.26%, which may be related to the thermal decomposition of the polysaccharide. The third weightlessness range occurs at 225–300 °C; due to the thermal decomposition of carbon, its mass gradually decreases. The fourth weightlessness range occurs at more than 300 °C, with a weightlessness value of 10.85%. Gao et al. [8] described a study in which the first thermostatic process that both types of polysaccharides exhibit was due to dehydration. The last two phases are due to a complex destruction process involving the dehydration and depolymerization of sugar rings to produce water and carbon dioxide.

For ASPS-SENPS, the degradation was roughly divided into two processes. The first stage of weight loss ranged from 10 to 250 °C, mainly due to the evaporation of water, with a weight loss of 36.80%, and its peak was at 177.6 °C; the second stage of weight loss ranged between 250 °C and above, with a mass loss of about 15.28%, which may be due to the loss of mass due to the thermal decomposition of polysaccharides.

### 2.7. IR Analysis

**Figure 9:**
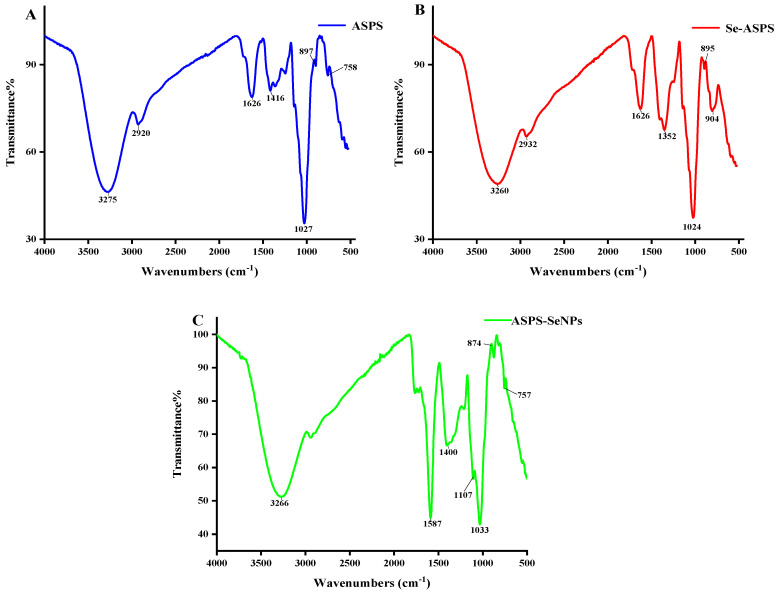
Infrared analysis. Note: (**A**) shows the IR spectral analysis of ASPS; (**B**) shows the IR spectral analysis of Se-ASPS; (**C**) shows the IR spectral analysis of ASPS-SeNPS.

The characterization of official groups in ASPS was analyzed using infrared spectroscopy. At 3275 cm^−1^ (ASPS), 3260 cm^−1^ (Se-ASPS), and 3266 cm^−1^ (ASPS-SENPS), the distinct and wideband is attributed to the stretched hydroxyl radicals (as illustrated in Figure 9). The spectral bands at 2920 cm^−1^ (ASPS) and 2932 cm^−1^ (Se-ASPS) were designated as stretching oscillations of C-H bonds [18]. In particular, the spectral bands at 1626 cm^−1^ (ASPS, Se-ASPS) and 1587 cm^−1^ (ASPS-SENPS) were designated as water-bonding vibrations [19]. The bending oscillations of the C-H bonds caused changes at 1416 cm^−1^ (ASPS), 1430 cm^−1^ (Se-ASPS), and 1400 cm^−1^ (ASPS-SENPS) [1]. A comparison of the infrared spectra of ASPS with Se-ASPS revealed a new absorptive peak at 904 cm^−1^ in Se-ASPS, which was ascribed to the asymmetric elongation of Se=O [7]. These results suggested that the selenization of ASPS occurred; comparing the infrared spectra of ASPS with those of ASPS-SENPS, the absorption bands are almost identical, suggesting that the backbone structure of ASPS remained unchanged after the formation of SeNPs. It is worth noting that the strong absorption of ASPS-SENPS at 3266 cm^−1^ is attributed to the O-H stretching vibration, which occurs at 3275 cm^−1^ for ASPS, suggesting that SeNPs preferentially formed Se-O bonds by interacting with OH groups [20]. This result also indicates that polysaccharides with O-H groups can bind to SeNPs [21].

### 2.8. Congo Red Test

**Figure 10:**
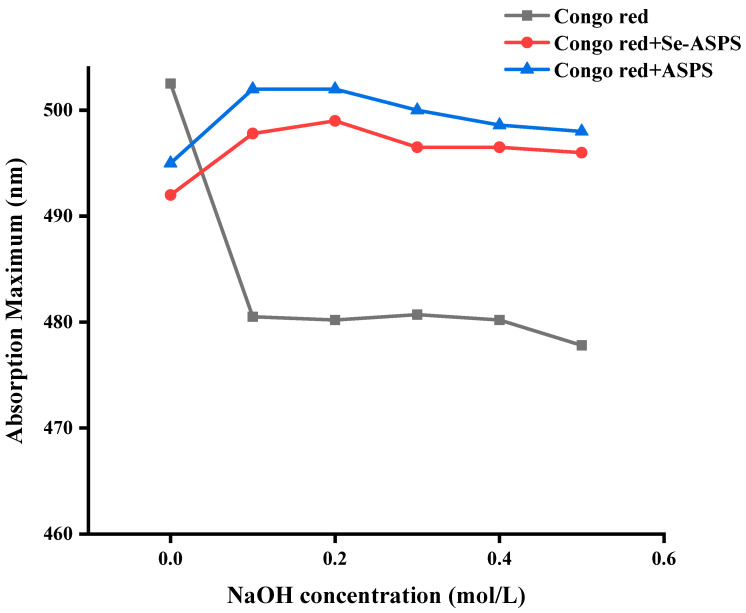
Congo red test.

Congo red can form a complex with polysaccharides with triple-helix structures in aqueous solution, and the maximum absorption wavelength of the complex will change according to the concentration of the NaOH solution: When the concentration of NaOH is low, λmax will move in the direction of the long wave; as the concentration increases, the hydrogen bonds in the triple-helix structure of the polysaccharides are destroyed, accompanied by deconvolution, irregular curls, and other conformational changes that may occur. At this time, λmax decreases, which produces the phenomenon of redshift. As can be seen from Figure 10, the ASPS and Se-ASPS curves have the same trend regarding λmax when the NaOH concentration increases, and there is a competitive relationship with the hydroxide ions in NaOH, resulting in different hydrolysis degrees of ASPS and Se-ASPS, which produces a change in the conformation of polysaccharide molecules in the aqueous solution. When the NaOH concentration is lower than 0.1 mol/L, ASPS and Se-ASPS just starts to form a complex with Congo red solution, at this time, the hydrogen bond of the triple-helix structure may not be destroyed to a large extent, and the λmax increases. In the range of 0.1~0.3 mol/L, the λmax of ASPS and Se-ASPS underwent fluctuations, which indicated that, at this time, ASPS and Se-ASPS may be experiencing the conformational change of triple helix → single helix → single helix → single helix → irregularly coiled. This indicates that both ASPS and Se-ASPS may be undergoing the conformational change of triple helix → single helix → irregularly curled, which leads to λmax changing unstably. Between 0.3 and 0.5 mol/L, the range of λmax of ASPS and Se-ASPS is not large, and it can be inferred that the triple-helix structure of polysaccharide has basically formed a complex with all of the Congo red at this time. It has also been reported that although chemical modification reduces the molecular mass of β-glucan, the structure of the glycan chain remains linked by β-1,3 glycosidic bonds due to the control of the reaction process of the triple-helix deconvolution to a single chain.

### 2.9. I_2_-KI Assay

**Figure 11:**
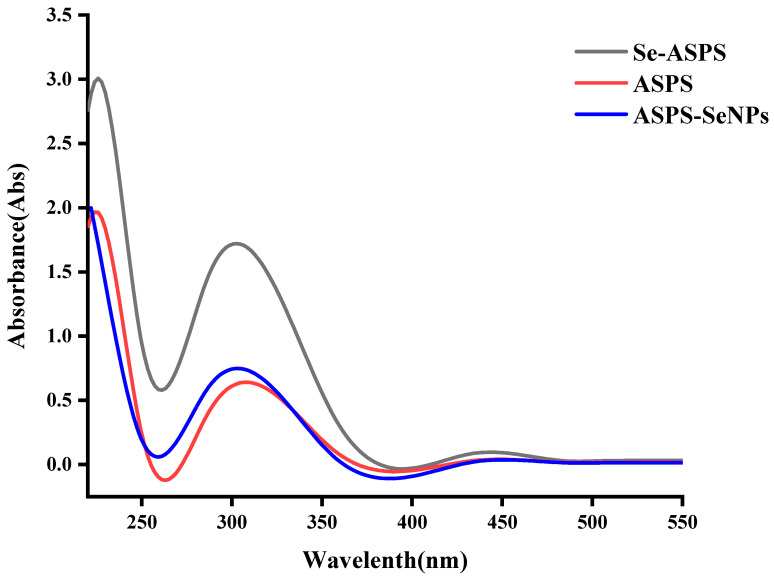
I_2_-KI test.

ASPS, Se-ASPS, and ASPS-SENPS showed no color reaction with I_2_-KI reagent and no absorption peaks at 500–700 nm in the UV spectrum, as shown in Figure 11. Starch is a polymer carbohydrate; the basic unit of starch is D-glucose, a polymer formed when glucose removes water molecules and is linked together by glycosidic bonds. There are two kinds of starch: straight-chain starch and branched-chain starch. Straight-chain starch is a polymer formed by connecting α-D-glucose glucose units through 1,4-glycosidic bonds, and the spatial conformation is curled into a helix [22]. Branched-chain starch molecules have α-(1,4)-glycosidic bonded sugar chains in addition to the presence of α-(1,6)-glycosidic bonded branches, and each branch is also curled into a helical shape. Starch is usually a mixture of linear and rectilinear amylopectin. The ability of iodine to work with starch to form a color is caused by the iodine molecules entering the spiral loops of starch to form a complex of starch and iodine. Branched-chain starch forms a brownish–red complex with iodine. Straight chain starch forms a blue complex with iodine. The maximum absorbance of straight chain starch and branched chain starch solutions with iodine is around 630 nm and 550 nm, respectively [23]. In this paper, ASPS, Se-ASPA, and ASPS-SENPS showed no color reaction with I_2_-KI reagent and no peaks of absorption from 500 to 700 nm in the UV pattern, which indicated that despite the glycosidic bonding of ASPS, Se-ASPA is dominated by the 1,4 linkage, and the spatial structure of ASPS, Se-ASPA is different from that of starch.

### 2.10. In Vitro Antioxidant Assay

**Figure 12:**
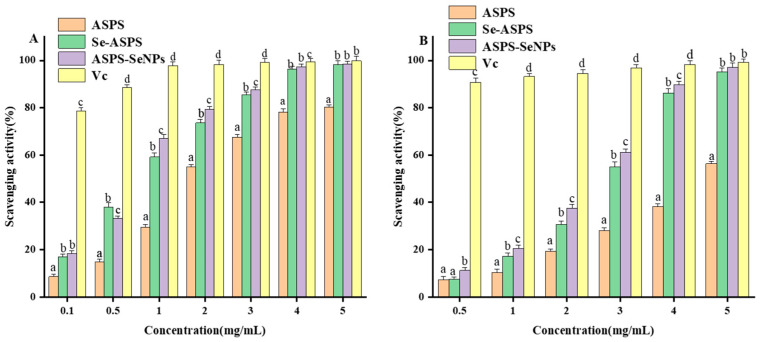
In vitro antioxidant tests of ASPS, Se-ASPS, and ASPS-SENPS: (**A**) ABTS free radical scavenging activity; (**B**) DPPH free radical scavenging activity. Note: Results labeled with the same letter are not significant; results labeled with different letters are significant. *p* < 0.05.

ABTS free radicals can be transformed into non-free radicals by receiving electrons from antioxidants [24]. The radical scavenging activity of ABTS free radicals by ASPS is illustrated in Figure 12A. ASPS, Se-ASPS, and ASPS-SENPS showed concentration-dependent scavenging. The radical scavenging activity of ABTS free radicals by Se-ASPS and ASPS-SENPS was significantly was higher than that of ASPS. At 5.0 mg/mL, the scavenging rates of ASPS, Se-ASPS, ASPS-SENPS, and ascorbic acid were 80.08%, 96.77%, 97.12%, and 98.20%, respectively, with IC50 values of 0.124, 0.062, and 0.057 mg/mL, respectively. At 4.0 mg/mL–5.0 mg/mL, the ability of Se-ASPS and ASPS-SENPS to scavenge ABTS was close to that of ascorbic acid.

Antioxidant substances are capable of scavenging DPPH free radicals; hence, the DPPH method is frequently used to study the antioxidant activity of these natural complexes [7]. The scavenging activities of ASPS and ascorbic acid against DPPH free radicals at different concentration levels are presented in Figure 12B. ASPS, Se-ASPS, and ASPS-SENPS DPPH free radicals showed significant scavenging activities in a concentration-dependent manner. The IC50 values of ASPS, Se-ASPS, and ASPS-SENPS were 0.947, 0.538, and 0.485 mg/mL. The results showed that the DPPH removal ability of Se-ASPS and ASPS-SENPS was not much different from that of ASPS at low concentrations, but with the increase in concentration, their DPPH removal ability was obviously much higher than that of ASPS. At a consistency of 5.0 mg/mL, the scavenging rates of ASPS, Se-ASPS, ASPS-SENPS, and ascorbic acid were 56.89%, 88.44%, 90.11%, and 99.56%, respectively, for DPPH radicals. Similar to the clearance of ABTS, the clearance of all three polysaccharides was lower than that of ascorbic acid at different concentrations, but at a density of 5.0 mg/mL, the clearance was similar to that of ascorbic acid. These results suggest that selenide-modified polysaccharides scavenge DPPH and ABTS radicals better than natural ASPS, which is consistent with previous findings that selenopolysaccharides have higher antioxidative potential [25,26]. The research found that the antioxidative activity of molecules depends on their ability to supply hydrogen. Selenate (-OSeO_2_H) [27] activates the hydronium substituent of the end-group carbohydrates in Se-ASPS. This accounts for why Se-ASPS shows superior antioxidant potential to ASPS.

## 3. Materials and Methods

### 3.1. Drugs and Chemical Reagents

Primary materials: *A. senticosus* was purchased from a local market. The other materials we used are as follows: sodium selenite (Sinopharm Group Chemical Reagent Co., Ltd., Shanghai, China); toluene (Yantai Far East Fine Chemical Co., Ltd., Yantai, China); nitric acid (Sinopharm Group Chemical Reagent Co., Ltd., Shanghai, China); potassium iodide (Shanghai Maclean’s Biochemistry Science and Technology Co., Ltd., Shanghai, China); phenol (Shanghai Maclean’s Biochemistry Science and Technology Co., Ltd., Shanghai, China); DEAE-52 cellulose (Shanghai Yuanye Biological Ltd., Shanghai, China); o-phenylenediamine (Shanghai McLean Biochemical Technology Co., Ltd., Shanghai, China); ascorbic acid (Shanghai Aladdin Biochemical Technology Co., Ltd., Shanghai, China); and EDTA (Sinopharm Chemical Reagent Co., Ltd., Shanghai, China).

### 3.2. Instruments and Equipment

Magnetic stirrer, JB-1A, Precision Scientific Instrument Co., Ltd., Shanghai, China; DK-8D, Huitai Instrument Manufacturing Co., Ltd., Foshan, China; vacuum-drying oven, model dZf-6050, Huitai Instrument Manufacturing Co., Ltd., Foshan, China; visible spectrophotometer, W14566722, Shanghai Xinmao Instrument Co., Ltd., Shanghai, China; swing-type high-speed grinder, DFY-210 multi-purpose, Zhejiang Dalin Machinery Co., Ltd., Zhejiang, China; ultrasonic cleaning machine, SB25-12, Xinzhi Biotechnology Co., Ltd., Zhejiang, China; low-speed bench centrifuge, TDZ6-WS, Xiangyi Experimental Instrument Co., Ltd., Xiangtan, China; blast Dryer, DHG-9140, Yiheng Scientific Instrument Co., Ltd., Shanghai, China.

### 3.3. Methods

#### 3.3.1. Preparation of Crude Polysaccharides of *A. senticosus*

##### Extraction and Purification of ASPS

According to the results of the pre-experiment, the specific method for the preparation of *A. senticosus* was as follows: the *A. senticosus* were beaten into powder form with a grinder, and 1 g of *A. senticosus* powder was weighed and placed in a conical flask; 10 mL of 0.3 mol/L hydrochloric acid solution was added, and it was placed in hot water of 90 °C for three hours and removed. Then, it was released to room temperature, and subsequently supplemented with 10 mL of 0.3 mol/L sodium hydroxide solution to neutralize the solution. After centrifugation to remove the *A. senticosus* powder in the solution, alcohol precipitation took place. After alcohol precipitation, it was placed in a drying oven at 60 °C. After drying, the *A. senticosus* polysaccharide powder was obtained.

In this experiment, the purification of ASPS was performed by the Sevage method, which has a good deproteinization effect. Chloroform and n-butanol were used in proportions of 4:1. These were added to the sample, which was shaken well to denature the proteins in the example polysaccharides to an insoluble state. Then, the proteins were removed by centrifugation. Hydrogen peroxide was used for decolorization, and hydrogen peroxide solution was added to the already deproteinated ASPS solution until its content was 5%. The solution was put into a water bath at 80 °C for 30 min before being removed and allowed to cool. Using the cellulose DEAE-52 column layer analysis method to elute the gradient of polysaccharides, we performed eluting with double-distilled water (ddH_2_O) until no sugar fraction came out and then performed eluting with NaCl. The concentration of NaCl was 0.1, 0.5 mol·L^−1^, speed of flow 1.0 mL·min^−1^; the control collector was 10 mL/tube. Then, the sugar content was measured. The results were plotted on the standard curve and combined with the corresponding tube numbers.

#### 3.3.2. Preparation of ASPS-SENPS

A total of 20 mL of 0.1 mol/L ascorbic acid solution was placed in a conical flask; 10 mg/L of spikenard polysaccharide solution was added, and 10 mL of 0.05 mol/L sodium selenite (Na_2_SeO_3_) solution was added. The flask was shaken well and then placed in a thermostatic water bath for heating.

- Optimization of preparation conditions for ASPS-SENPS.
- Effect of ASPS addition

Ascorbic acid solution (20 mL 0.1 mol·L^−1^) was put into an Erlenmeyer flask. We then added 10 mL *A. senticosus* polysaccharide solution with concentrations of 7, 8, 9, 10, 11, and 12 mg/L, respectively, and then added 10 mL 0.05 mol·L^−1^ of sodium selenite (Na_2_SeO_3_) suspension. The mixture was shaken well and then placed in a thermostatic water bath before being heated for 8 h. The effect of the amount of *A. senticosus* polysaccharide on the synthesis of ASPS-SENPS was investigated.

- Effect of reaction temperature

Ascorbic acid (20 mL 0.1 mol·L^−1^), *A. senticosus* polysaccharides (10 mL, 10 mg/L), and sodium selenite (10 mL, 0.05 mol·L^−1^) were transferred into a conical flask, which was sufficiently shaken and then transferred into a thermostatic water bath (30, 40, 50, 60, 70, 80, and 90 °C), where it stayed for 8 h, to investigate the effect of reaction temperature on the synthesis of ASPS-SENPS.

- Effect of reaction time

Ascorbic acid solution (20 mL 0, 1 mol/L) was put into a conical bottle. We then added 10 mL 10 mg/L *A. senticosus* polysaccharide solution and then added 10 mL 0.05 mol·L^−1^ sodium selenite (Na_2_SeO_3_) solution. After being shaken well, the reaction was placed in a constant-temperature water bath for 1, 2, 4, 6, 8, and 10 h. The effect of reaction temperature on the synthesis of ASPS-SENPS was investigated.

- Effect of Ascorbic acid–Na_2_SeO_3_

Ascorbic acid solution (0.1 mol/L) was pipetted into a conical bottle, and 10 mL of 10 mg/L *A. senticosus* polysaccharide solution was added. Then, sodium selenite (Na_2_SeO_3_) solution was added to make ascorbic acid–sodium selenite (1:1, 2:1, 3:1, 4:1, 5:1, and 6:1). After being shaken well, the reactants were put into a thermostatic water bath for 8 h. The effects of the ratios of ascorbic acid and sodium selenite on the synthesis of nanosized selenium from *Erythrina* polysaccharides were investigated.

#### 3.3.3. Preparation of Se-ASPS

- Determination of Selenium Content

Absorbance was measured as the vertical coordinate by UV-vis spectrophotometry, with the mass concentration of selenium as the horizontal coordinate according to the description in [28].

Preparation of selenium standard solution: Selenium standard reserve solution (100 μg/mL): 100.0 mg elemental selenium (spectral pure) was accurately weighed, dissolved in a small amount of nitric acid, supplemented with 2 mL perchloric acid, heated in boiling water thermostatted for 3–4 h, supplemented with 8.4 mL hydrochloric acid after cooling, and then cooked in a boiling water bath for 2 min. It was accurately diluted to 1000 mL, and its hydrochloric acid concentration was 0.1 mol/L. The reserve liquid concentration was 100 μg/mL.

Selenium standard solution (5 μg/mL): We diluted the above 100 μg/mL selenium solution with 0.1 mol/L hydrochloric acid so that the selenium content was 5 μg/mL and stored it in the refrigerator (we added 100 μg/mL 5 mL selenium solution to 90 mL 0.1 mol/L hydrochloric acid solution, that is, the required selenium solution). A certain amount of selenium standard solution (0.0, 1.0, 2.0, 3.0, 4.0, 5.0, 6.0, 7.0, and 8.0 mL) was accurately removed and added into 8 conical bottles; 1 mL 0.2 mol/L EDTA solution and 2 mL 0.02% o-phenylenediamine solution were added, shaken, and left for 1 h. We removed the liquid, added water to 35 mL, and then added 10 mL toluene. The mixture was shaken for 3 min and left to stand for 10 min, and then we transferred the extract of the toluene layer into a 1 cm quartz colorimetric dish, using toluene as a reference, and the absorbance at 334 nm was determined. The standard curve equation A = 0.0214x + 0.0051, R^2^ = 0.9993, was fitted, indicating good linearity from 0 to 80 μg·mL^−1^. A certain amount of selenium polysaccharide sample of *A. senticosus* was accurately weighed and processed according to the method described in [28], and then the selenium content in Se-ASPS was calculated by substituting the standard curve equation.

- Preparation of Se-ASPS

We adopted the HNO_3_-Na_2_SeO_3_ method [12], weighed ASPS, and put it into a conical flask. We slowly added the volume fraction, 0.5% nitric acid solution, stirring while adding it. After dissolution, Na_2_SeO_3_ and BaCl_2_ were added, the temperature of the water bath was adjusted to 80 °C, and the reaction was kept constant for a particular time. At the end of the reaction, it was cooled to 25 °C, and we adjusted the pH to 7 with NaOH (1 mol·L^−1^) solution. We added a certain amount of Na_2_SO_4_ to remove Ba^2+^, centrifuged the mixture, and dialyzed the supernatant (the relative molecular weight was cut off at 8000–10,000) until there was no selenium (we took a small amount of dialysate and tested with ascorbic acid until the test solution had no red color), and then obtained *A. senticosus* polysaccharides.

- Optimization of Preparation Technology of Selenium Polysaccharide of *A. senticosus*
- Effect of reaction temperature

We weighed 500 mg of *A. senticosus* polysaccharide and placed it in an Erlenmeyer flask. We slowly added 50 mL nitric acid solution with a volume fraction of 0.5%, stirring the mixture while this was added. After dissolution, 400 mg of Na_2_SeO_3_ and an appropriate amount of BaCl_2_ as a catalyst were added; the mixture was left at a constant temperature for 9 h. Then, we proceeded to examine the effect of reaction temperature on selenium content at 30, 40, 50, 60, 70, and 80 °C.

- Effect of reaction time

We weighed 500 mg of *A. senticosus* polysaccharide put it into a conical flask; then, nitric acid (50 mL, 0.5%) was added slowly dropwise, with the mixture being stirred while adding the nitric acid. After dissolution, 400 mg of Na_2_SeO_3_ and an appropriate amount of the BaCl_2_ catalyst were put in, and the reaction was carried out at a controlled temperature of 60 °C to investigate the effect of reaction time at 7, 8, 9, 10, 11, and 12 h on the selenium content.

- Effect of sodium selenite dosage

We weighed 500 mg *A. senticosus* polysaccharide and placed it in an Erlenmeyer flask. We slowly added 50 mL nitric acid solution with a volume fraction of 0.5% and stirred the mixture while adding the nitric acid solution. After dissolution, we added Na_2_SeO_3_, and the appropriate amount of the BaCl_2_ catalyst; the reaction was carried out at a temperature of 60 °C for 11 h. The effects of Na_2_SeO_3_ addition on Se content were investigated at 100, 200, 300, 400, 500, and 600 mg concentrations.

- Orthogonal test of the preparation process of Se-ASPS

In order to obtain a higher content of Se-ASPS, nine modeling conditions were designed based on a three-factor L_9_(3^4^) orthogonal test by using the results of a one-factor test, and then the sodium selenite dosage, the response temperature, and the response time were used as the influencing factors, which were 400, 500, 600 mg, and 500 mg of ASPS for Na_2_SeO_3_ and the reaction temperatures of 40, 50, and 60 °C. Reaction times of 9, 10, and 11 h were used (Table 3). We added 0.5 g of ASPS to a 0.5% HNO_3_ solution and stirred it until it completely dissolved. The conditions for the orthogonal experiment are shown in Table 3. At the end of the reaction, the solution was cooled down to room temperature, and the pH was regulated to perform orthogonal tests and determine the selenium content in Se-ASPS.

#### 3.3.4. Chemical Composition Analysis

The protein content, polysaccharide content, selenium content, glyoxylate content, and carbohydrate content of ASPS, Se-ASPS, and ASPS-SENPS were determined experimentally.

#### 3.3.5. Ultraviolet–Visible Spectral Profiling

Distilled water solutions (2 mg/mL) of ASPS, Se-ASPS, and ASPS-SENPS were determined using a microspectrophotometer (Shimadzu-uv-2550, Shimadzu Co., Kyoto, Japan). Its UV spectrum was in the range of 200–800 nm.

#### 3.3.6. Characterization

- Scanning Electron Microscopy (SEM)

We weighed an appropriate amount of the ASPS, Se-ASPS, and ASPS-SENPS; after loading them into the sample platform, plating was performed. The topographic structure of the polysaccharides was then scratched using a field emission scanning electrochemical microscope (FESEM, S4800, Hitachi Limited, Tokyo, Japan). The operational conditions were a high pressure of 5.0 kV, operating separation of 5.4 mm, and a magnification of 20.0 K. The results were summarized in the following table.

- Thermogravimetric Analysis (TGA)

Thermogravimetric analysis of ASPS, Se-ASPS, and ASPS-SENPS was performed using a thermal analyzer (STA 449C, NETZSCH, Selb, Germany) to determine their thermogravimetric properties when heated at a rate of 10 °C/min between 25 °C and 900 °C with a streaming rate of 65 mL/min of nitrogen.

- Analysis of Fourier Transform Infrared (FT-IR)

Following drying in an incubator for 2 h, 2.0 mg of the samples of ASPS, Se-ASPS, and ASPS-SENPS were, respectively, mixed with 100–200 mg of dry kammonium bromide (KBr), ground in a marcasite mortar, and pulverized into flakes using the KBr pelleting technique. Their FT-IR spectra were screened in the wave number range of 4000–400 cm^−1^ using a TENSOR27 FT-IR microspectrometer (Brooker, Saarbrucken, Germany).

- Congo Red Test

ASPS, Se-ASPS, and ASPS-SENPS solutions (1 mg/mL) were mixed with Congo red mixture (80 mol/L), respectively, and we then applied NaOH (1 mol/L) to the mixture and adjusted the NaOH density (0, 0.1, 0.2, 0.3, 0.4, and 0.5 mol/L). The maximum absorption spectra were recorded using a UV-vis spectrophotometer from 400 to 700 nm for the maximum absorption peak of the mixture. The maximum absorption wavelength (λmax) of the sample was plotted against the λmax of Congo red in the NaOH solutions.

- Potassium Iodide Test (I_2_-KI)

A polysaccharide solution of 1 mg/mL was used to prepare pure water, and 2 mL of ASPS, Se-ASPS, and ASPS-SENPS solutions were mixed thoroughly with 1.2 mL of I_2_-KI solution (0.02% from I_2_ and 0.2% from KI). Color changes were observed, scanned, and analyzed using an enzyme standardizer in the wavelength range of 300–700 (at an interval of 5 nm).

#### 3.3.7. In Vitro Antioxidant Activity Test

- DPPH radical scavenging activity

Referring to the research method of Wang et al. [29], 2 mL of the sample solution (concentration gradient: 0. 5, 1, 2, 3, 4, and 5 mg/mL) and 4 mL DPPH solution were evenly mixed and left to stand in the dark for 30 min. The absorbance value was detected at 517 nm, and clearance and half inhibitory concentration (IC_50_) were calculated. Absolute ethanol was used as the blank control, and L-ascorbic acid of the same mass concentration was employed as a positive response.

$$\mathrm{DPPH}\;\mathrm{free}\;\mathrm{radical}\;\mathrm{clearing}\;\mathrm{rate}=\frac{A_{0}-(A_{1}-A_{2})}{A_{0}}\;\times \;100\%$$

where *A*_0_ is the control group (anhydrous ethanol + DPPH absorbance); *A*_1_ is the experimental group of the sample + DPPH); and *A*_2_ is the background group of the sample (sample + anhydrous ethanol absorbance).

- ABTS radical scavenging activity

A modified version of the method of N J Miller et al. [30] was used to determine the removal activity of ABTS radicals by ASPS before and after the modification. Then added 7 mM ABTS solution and 2.5 mM of potassium peroxydisulfate solution, mixed in equal volumes, and then the mixture was left to stand at room temperature for 14 h and protected from light to obtain the ABTS reserve solution. Before use, the ABTS reserve solution was dissolved in 75% ethanol, and the absorbance value at 734 nm was 0.7 ± 0.02. Next, 0.2 mL of ASPS extract before and after modification was added to 5.8 mL of ABTS working solution with the following concentration gradient: 0.5, 1, 2, 3, 4, and 5 mg/mL. Then, the reaction was carried out for 10 min. Absorbance values were measured at 734 nm, and the clearance and half inhibitory concentrations (IC_50_) were calculated. Anhydrous ethanol was used as a blank control, and the same mass concentration of L-ascorbic acid was used as a positive control.

$$\mathrm{ABTS}\;\mathrm{free}\;\mathrm{radical}\;\mathrm{scavenging}\;\mathrm{rate}=\frac{A_{0}-(A_{1}-A_{2})}{A_{0}}\;\times \;100\%$$

where *A*_0_ is the control group (anhydrous ethanol + ABTS); *A*_1_ is the experimental group of the sample + ABTS); and *A*_2_ is the sample background group (sample + anhydrous ethanol absorbance).

### 3.4. Statistical Analysis

All figures were produced using the Microsoft Excel (Office 2013) and Origin programs version 9.6. One-way analysis of variance (ANOVA) was used to analyze the data, and then Scheffe’s multiple correlation analysis was performed using IBM SPSS 18.0. The level of significance (*p*) was established at 0.05.

## 4. Conclusions

Many studies have found that appropriate molecular modifications or structural modifications can lead to new activities or the further enhancement of the original activities of polysaccharides [31]. The selenization modification of polysaccharides has become a focus of attention. Both elemental selenium and polysaccharides have unique functional properties, and both can exert their respective pharmacological effects in animal organisms. Selenopolysaccharide is the product of combining selenium with polysaccharide in organic form, which has the dual effects of selenium and polysaccharide. Synthetic selenopolysaccharides are obtained by extracting natural polysaccharides and then chemically reacting the polysaccharides with selenium to obtain selenium-modified polysaccharides. Among the synthetic methods, the nitrate–sodium selenite method is widely used for the synthesis of selenopolysaccharides because of its easy operation and economic efficiency. Hou et al. [32] synthesized *Lily* selenized polysaccharide with a high selenium content of 41.30 mg/g using the nitrate–sodium selenite method. Sun et al. [33] synthesized *Ornithogalum caudatum* Ait. (Liliaceae) selenized polysaccharide with a selenium content of 3.125 mg/g using the nitrate–sodium selenite method. Shao et al. [12] successfully prepared two kinds of selenized polysaccharides of blue Lonicera japonica fruit using the nitrate–sodium selenite method with selenium contents of 228 ± 24 and 353 ± 36 μg/g, respectively. In the present study, selenized Lonicera japonica polysaccharides with a selenium content of 145.78 μg/g were obtained by adding 600 mg of Na_2_SeO_3_ and an appropriate amount of BaCl_2_ as a catalyst in acidic conditions and carrying out a reaction at a constant temperature of 60 °C for 9 h. The selenium content of *A. selenopanax* selenized polysaccharides was 145.78 μg/g, which was obtained by using the nitrate–sodium selenite method.

The use of selenium sources as nutritional supplements or additives has become a pressing issue. Selenium nanoparticles (SeNPs) are red elemental selenium with extremely small particles that can be absorbed by the body and are biologically active. Compared with other inorganic or organic forms of selenium sources, SeNPs have higher bioactivity and lower toxicity. SeNPs have become a research hotspot in recent years due to their tiny size and special physicochemical properties, and their production methods, characteristics, and potential applications in life sciences have attracted great attention. However, due to the high surface energy of SeNPs [34], they are generally unstable in aqueous solution, which severely limits their practical applications. Therefore, some biomolecules, including polyphenols [35], proteins [36], and especially polysaccharides, have been used as stabilizers and modifiers to prepare spherical nano-selenium to improve the stability of nanocomposites [37]. In addition, polysaccharides are biomolecules with multiple bioactivities [38] and have higher energy efficiency in SeNP decoration compared to proteins and polyphenols [39]. Jiao et al. successfully prepared nano-selenium using *Astragalus* polysaccharide, and their prepared *Astragalus* polysaccharide–nano-selenium had a stability of 35 d at 4 °C. In vitro anti-hepatocellular carcinoma experiments also showed that astragalus polysaccharide–nano-selenium significantly inhibited the proliferation of HepG2 cells in a dose-dependent manner; Gao et al. successfully prepared nano-selenium using *Berberidis radix* polysaccharide and demonstrated that *Berberidis radix* polysaccharide–nano-selenium could increase the cell viability and GSH-Px enzyme activity of the cellular damage model induced by H_2_O_2_ cell viability and GSH-Px enzyme activity and reduced MDA content. In this study, ASPS was used as a stabilizer and dispersant for nanocomposites (ASPS-SeNPs), which were prepared by modifying SeNPs, and the results showed that the optimal synthesis conditions were as follows: the amount of *A. selenopanax* polysaccharides was 100 mg, the reaction time was 6 h, the reaction temperature was 50 °C, and the ratio of ascorbic acid and sodium selenite was 4:1.

Scanning electron microscopy showed that the surface of Se-ASPS was uneven and relatively rough, with a large number of grooves, strong adhesion, and small irregular holes on the surface, which was due to the surface changes caused by intermolecular forces between selenium and polysaccharides, van der Waals forces. It has been reported that α-glycosidic bonds may connect selenium to monosaccharide residues in polysaccharides [40], and elemental selenium may be present in selenopolysaccharides in the form of C-O-SeO_3_ or Se=O, which alters the structure of the polysaccharide chain.

The results of in vitro antioxidant capacity showed significant differences between ASPS and ASPS-SeNPs and Se-ASPS. ASPS-SeNPs and Se-ASPS possessed strong antioxidant activities, including the scavenging of ABTS radicals and DPPH radicals. Both selenated polysaccharides showed better antioxidant activity compared to natural polysaccharides. It has been proposed that selenium in selenated polysaccharides mainly exists in the form of selenate groups [41]. The presence of these groups activates the hydrogen atoms of the head carbon and promotes its antioxidant activity [27]. In addition, the molecular weight of polysaccharides decreases after selenization due to the degradation of polysaccharide chains during selenization [42]. In general, low-molecular-weight polysaccharides have more reduced hydroxyl termini to accept and scavenge free radicals. SeNPs have been reported to have good in vitro free radical scavenging ability and antioxidant activity, which is closely related to the particle size and stability of the nanoparticles [14]. The smaller the particle size, the larger the relative specific surface area and the more sites for reaction with free radicals. In addition, SeNPs have good stability and a positive scavenging effect on free radicals. Poor stability in SeNPs leads to more nanoparticle aggregation and fewer surface reactions with free radicals.

In summary, the results of the present study, to some extent, resolved the structural characterization of selenated *A. selenopanax* polysaccharides and *A. selenopanax* polysaccharide–nano-selenium and compared their properties and biological activities, providing a certain theoretical basis for further research on *A. selenopanax* polysaccharides as drugs and functional foods, and the structural features of selenated *A. selenopanax* polysaccharides and *A. selenopanax* polysaccharides-nano-selenium will be further resolved at a later stage, while the mechanisms of the antioxidant effects of selenated *A. selenopanax* polysaccharides and *A. selenopanax* polysaccharide–nano-selenium will be further analyzed. At the same time, we will further analyze the antioxidant mechanism of selenized *A. selenopanax* polysaccharides and *A. selenopanax* polysaccharide–nano-selenium.

## Figures and Tables

**Table 1:** Modification conditions and selenium contents of Se-ASPS.

	Na_2_SeO_3_ (mg)	Temperature (°C)	Time (h)	Selenium Content (mg/g)	Carbohydrate Content (%)
sASPS_1_	600	60	9	145.78	69.53
sASPS_2_	400	50	11	140.92	61.21
sASPS_3_	600	40	11	138.3	58.01
sASPS_4_	400	60	10	110.63	61.21
sASPS_5_	500	60	11	121.48	75.61
sASPS_6_	600	50	10	99.98	72.09
sASPS_7_	500	50	9	91.76	80.73
sASPS_8_	500	40	10	107.83	73.69
sASPS_9_	400	40	9	114.56	80.41

**Table 2:** Analysis of the chemical composition of *A. senticosus* polysaccharides before and after selenization modification.

Item	Selenium Content (mg/g)	Protein Content (%)	Glucuronic Acid Content (%)	Polysaccharide Content (mg/g)	Reducing Sugar Content (%)
Se-ASPS	145.78 ± 3.20	1.60 ± 0.08	2.31 ± 0.49	17.12 ± 1.01	49.36 ± 1.51
ASPS	5.34 ± 0.12	4.30 ± 0.51	3.04 ± 0.16	11.47 ± 1.82	13.60 ± 1.82
ASPS-SENPS	21.92 ± 2.01	7.91 ± 1.15	3.50 ± 1.04	14.29 ± 1.38	42.13 ± 0.53

**Table 3:** Orthogonal experimental design.

Level	Dosage of Sodium Selenite (mg)	Temperature of the Reaction (°C)	Duration of the Reaction (h)
1	400	40	9
2	500	50	10
3	600	60	11

## Data Availability

Data will be made available on request.
